# In-field assessment of change-of-direction ability with a single wearable sensor

**DOI:** 10.1038/s41598-023-30773-y

**Published:** 2023-03-18

**Authors:** Salil Apte, Hojjat Karami, Célestin Vallat, Vincent Gremeaux, Kamiar Aminian

**Affiliations:** 1 Laboratory of Movement Analysis and Measurement, 1015 Lausanne, Switzerland; 2 Institute of Sport Sciences, University of Lausanne, Lausanne, Switzerland; 3 Sport Medicine Unit, Division of Physical Medicine and Rehabilitation, Swiss Olympic Medical Center, Lausanne University Hospital, Lausanne, Switzerland

**Keywords:** Electrical and electronic engineering, Translational research

## Abstract

The Agility T-test is a standardized method to measure the change-of-direction (COD) ability of athletes in the field. It is traditionally scored based on the total completion time, which does not provide information on the different CODs. Augmenting the T-test with wearable sensors provides the opportunity to explore new metrics. Towards this, data of 23 professional soccer players were recorded with a trunk-worn GNSS-IMU (Global Navigation Satellite System-Inertial Measurement Unit) device. A method for detecting the four CODs based on the wavelet-denoised antero-posterior acceleration signal was developed and validated using video data (60 Hz). Following this, completion time was estimated using GNSS ground speed and validated with the photocell data. The proposed method yields an error (mean ± standard deviation) of 0 ± 66 ms for the COD detection, − 0.16 ± 0.22 s for completion time, and a relative error for each COD duration and each sequential movement durations of less than 3.5 ± 16% and 7 ± 7%, respectively. The presented algorithm can highlight the asymmetric performance between the phases and CODs in the right and left direction. By providing a more comprehensive analysis in the field, this work can enable coaches to develop more personalized training and rehabilitation programs.

## Introduction

Agility is an important ability for athletes in sports like soccer, rugby, hockey, tennis, badminton, etc., as they are required to respond quickly to events on the field. In this context, the capacity to perform a "rapid whole-body movement with change of velocity or direction in response to a stimulus"^1^ is defined as agility. Agility is broadly based on two components: a reactive component involving cognitive factors as perception, reaction time, anticipation, etc., followed by an athletic component involving speed, acceleration, strength, coordination, technique etc., to execute the planned movement. Agility, being multifactorial, is difficult to evaluate quantitatively during in-field training and testing. However, the second component of agility, i.e., the ability to rapidly execute a pre-planned movement, can be evaluated using change-of-direction (COD) tests in the field. The most important factors affecting the technical execution of direction changes are the approach speed and the COD angle, where the latter is defined as the angle between the direction of body progression before and after the COD. While there is no “gold-standard" COD test, a "T" shape of ten yards^2^ is commonly used in sports such as soccer^3^, basketball^4^, football^5^, tennis^6^, etc. This test has been shown to be reliable for effectively measuring COD ability^2,7^. Furthermore, it is popular because of the ease of use—a trial usually takes less than 15 s, only cones are needed to mark the path and a pair of photocells to time it, and the test requires little space. Therefore, the T-test is the focus of this work.

Performance on the COD T-test is not biased by cognitive factors because the trajectory is known, and the athletes can plan their movement in advance. This makes the measurements more homogeneous in an elite athlete population and eliminates the necessity to measure variables such as reaction time and cognitive load, which are difficult to evaluate on the field. The required qualities to perform a COD are supposed to be a mix of: speed, balance, technique (anticipatory and proprioception skills), strength, power, plyometric capacity, etc.^1^. Therefore, COD speed tests are an effective way to assess multiple physical abilities at once in an environment closer to the field. Rapid performance of COD requires an extended knee position, greater knee abduction angles, and large ground reaction forces. Because these factors are related to knee joint strength and stability^8^, COD test is also used as a tool to assess return to sport in athletes with anterior cruciate ligament (ACL) injuries, which are a common prognosis in sports that require COD skills. The deterioration of athletes’ ability to decelerate rapidly before COD has been associated with loss of neuromuscular coordination, loss of strength, unconscious reduction of speed before COD for fear of re-injury, etc., and can therefore be detected by a detailed analysis of the COD test^9^.

Detailed analyses, however, are not performed very often in common practice and performance analysis is usually limited to the completion time. By using force plates, additional video analysis or wearable sensor technology, advanced metrics such as ground contact time during COD can be assessed^10^. Force plates can also be used to evaluate the magnitude of GRF, where high reaction force, especially during the braking phase before COD, correlates with better performance^11^. Complementing the force plates with a motion capture system can provide additional metrics such as speed before/after the COD, movement of the center of mass (COM), range of motion (ROM) of trunk rotation along mediolateral axis, knee adduction moment (KAM), etc.^12^. Furthermore, asymmetries of the range of motion of the shank between right and left side can be a way to measure knee stability^9^ and the movement of COM and the magnitude of GRF can be used to ascertain vertical stiffness^13^ during COD. While this measurement setup can enable the analysis of a large variety of metrics^14^, it is cumbersome to use during regular sessions on the field. In contrast, wearable sensor-based methods can provide an easier avenue for analysis in the field^15–19^ and have been previously used to augment functional capacity tests.

Wearable IMU systems have been utilized to analyze COD tests^20^ based on the changes in the body rotation about the vertical axis. As this method requires the athlete to look in the direction of running after COD, it is not applicable for the T-test. Another study used sacrum-worn IMU to distinguish between two different techniques used in a slalom run^21^ using a K-means clustering analysis. But the drift-reduction technique employed to assess the trunk orientation was not validated quantitatively. Machine learning approaches have also been employed to estimate running speed before/after turns using pelvis-mounted IMU^22^ and knee joint loading using an IMU each on the thigh and the shank^23^. However, the former work provided an association of R^2^ < 0.7 and only considered 180° cuts, while the latter study utilized a sleeve to attach the IMUs to the leg, thus making the estimation susceptive to soft tissue artefacts, especially during highly dynamics movements. While a quantitative analysis of the duration of each phase would be a beneficial for coaches to identify athletes’ weaknesses in a particular movement sequence or COD type, none of these works considered the T-test and only one study^20^ utilized a wearable sensor commonly used by high level athletes. Therefore, we aimed to develop a method that allows automatic detection of the different phases of a T-test as it can be a valuable tool for coaches. Thus, we designed algorithms for analyzing an instrumented T-test by a trunk-worn GNSS-IMU sensor. Furthermore, this work presents the validation of the duration of the detected phases and an initial exploration of the different performance metrics that can be estimated using this method.

## Methods

### Materials and protocol

**Figure 1:**
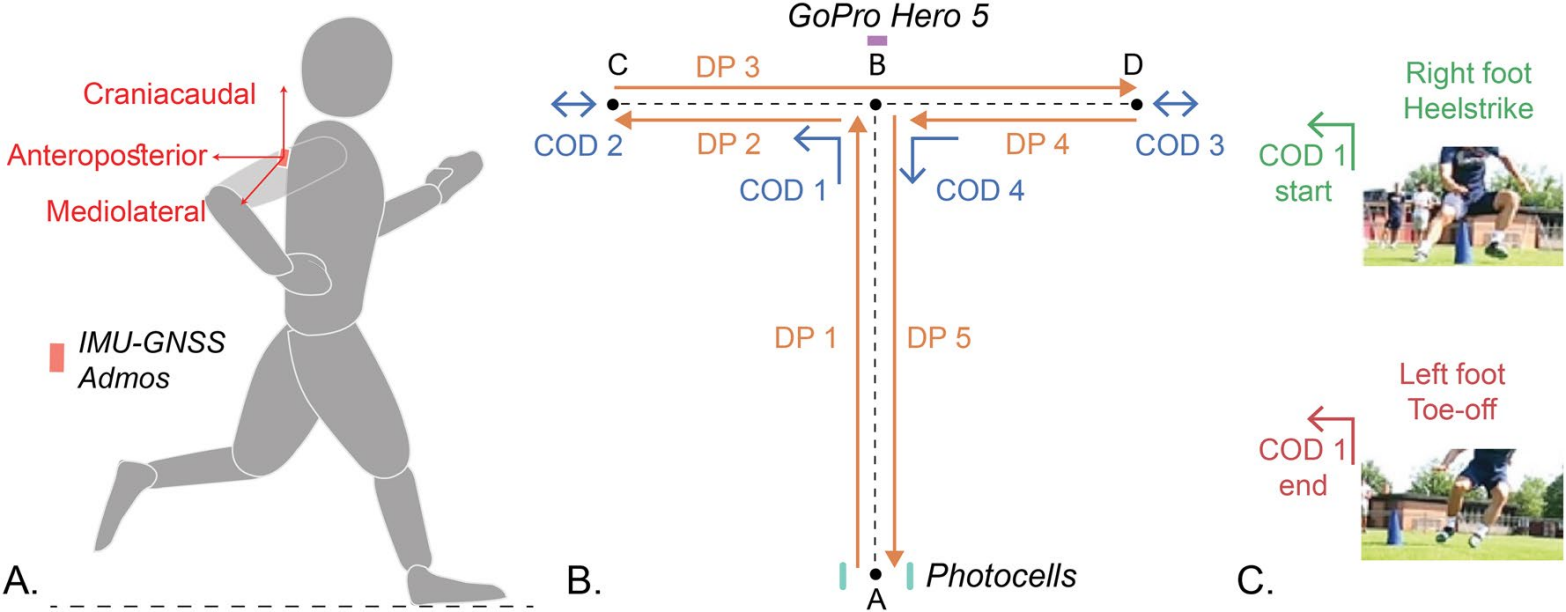
Sensor setup and the nine segments of the Agility T-test. Instrumentation used for the protocol is presented in Italics. For video labeling, start and end of COD 1 are defined as the right foot heel strike and left foot toe-off, respectively.

Twenty-three male professional soccer players (height: 181.4 ± 5.4 cm, weight: 75.0 ± 5.6 kg, age: 25.1 ± 4.3 years) from Swiss league football were enrolled in this study. The study was approved by the EPFL human research ethics committee (HREC 053-2020) and conducted in accordance with the declaration of Helsinki, and written informed consent was obtained from all the participants before the measurements. During actual preseason testing of the soccer team, each athlete underwent two trials of the standard T-test with instructions to give their best performance; only the second trial was with an IMU-GNSS sensor (AdMos, ASI, Switzerland) placed on the upper back (Fig. 1A). This sensor setup was chosen as it is commonly used in soccer^24^ and it was previously used for instrumenting a (30–60 m) straight-line sprint test^25^. Since the sprint test is typically carried out together with the T-test during pre-season testing in soccer, instrumentation of both tests using the same sensor setup can be valuable. The AdMos sensor was configured to measure 3D acceleration and angular velocity with the sampling frequency of 200 Hz as well as GNSS ground speed with the sampling frequency of 10 Hz. Video data of all tests were recorded with a camera (Gopro Hero 5, frame rate = 60 fps), pointed at the athlete and were used as reference for labelling the different phases of the test. The total completion time of the test was measured using a photocell (Witty, Microgate corp, Italy) placed at the start line. The photocell beeped when the athlete crossed the start line at the start and the end of the test. During the test, the athlete was instructed to face forward, touch each cone, and to not cross the feet when shuffling sideways. Each T-test was divided into 9 segments (Fig. 1B) that consist of 4 CODs and 5 displacement phases (DPs). During each COD, the athletes must touch the cones, indicated by points B, C, and D:

1. DP 1: Forward sprint from A to B which begins with the photocell sound
2. COD 1: 90° angle COD using sidestep, from forward sprint to sideways displacement in the left direction
3. DP 2: First left shuffle which is sideway displacement from B to C
4. COD 2: 180° angle COD using split-step, from left shuffle to sideways displacement in the right direction
5. DP 3: Right shuffle which is sideway displacement from C to D
6. COD 3: 180° angle COD using split-step, from right shuffle to sideway displacement in the left direction
7. DP 4: Second left shuffle which is sideway displacement from D to B
8. COD 4: 90° angle change of direction using sidestep, from left shuffle to backward sprint
9. DP 5: Backward sprint from B to A that ends with the second photocell sound.

### Labelling of video data

For validation of the segmentation algorithms, it is important to label each of defined segments from the recorded videos. For each COD segment, we defined five distinctive events: one HS (heel strike) each for RF (right foot) and LF (left foot), one TO (toe-off) each for RF and LF, and one for touching the cone. The COD motion can be divided into two main phases: the eccentric phase (braking) and the concentric phase (pushing). The transition between the two phases occurs approximately when the athlete touches the cone. Just before the eccentric phase and just after the concentric phase (of both legs), the athlete is no longer in contact with the ground. COD duration is the time the athlete is in contact with ground, after the first heel strike of the last flight phase of the approach displacement phase and the last toe-off of the first flight phase after the COD. Assigned events for each COD are depicted in the supplementary materials (Figure S2) and briefly described below:

- COD 1: Start and end are defined as the RF HS and LF TO, respectively (Fig. 1C)
- COD 2: Start and end are defined as the RF HS and RF TO, respectively
- COD 3: Start and end are defined as the LF HS and LF TO, respectively
- COD 4: Start and end are defined as the RF HS and LF TO, respectively.

Time frames of each test were recorded using Kinovea^®^ and then imported into MATLAB. Each test data was manually cut in a 15-s window (the duration of the T-test is typically around 10 s) for subsequent algorithm development and validation.

### Algorithm development

A macro–micro approach is followed to detect COD segments, which has already been successfully applied in biomechanical assessment using wearable IMUs^26^. First, a macro-analysis of the signal was performed to identify the beginning and end of the test as well as the transient phases corresponding to each of COD segments. Then, micro-analysis methods are were developed for a more accurate detection of step patterns in each COD segments. COD  detection algorithms were developed using data from 6 randomly selected athletes out of the total 23. Furthermore, we proposed an algorithm for estimating total completion time based on GNSS ground speed.

#### Macro-analysis

The reference for the beginning of the test was considered the time when the athlete crosses the start line and the corresponding beep was heard from the photocell. Due to lack of synchronization between photocell and the IMU, it was not possible to locate the exact timestamp of the beginning of the test on the IMU signal. Therefore, the video and the signal were synchronized based on the event of the first step. The sound of the photocell from the video was assumed to be the reference starting point while this first step was detected from the IMU signals. Acceleration in the anterior–posterior (AP) direction was used to determine the beginning of the test. While the axes were defined in the sensor frame (Fig. 1A), they were assumed to be aligned with the body frame due to the location of the sensor within the vest and the tight fit of the vest. Thus, we can neglect relative movement of the sensor with respect to the trunk. This signal was filtered using a zero-phase 2nd order Butterworth low-pass filter with a cut-off frequency of 10 Hz. The first two local maxima above 4 ms^−2^ were detected from this signal using the *peakfinder* function in MATLAB 2020b. The first peak likely originated from the straightening of the upper body as the athlete began to push on the ground whereas the second peak likely came from the impact during the first step of the test. Therefore, the local minimum before these two peaks was defined as the start of the movement and the first HS (first step).

**Figure 2:**
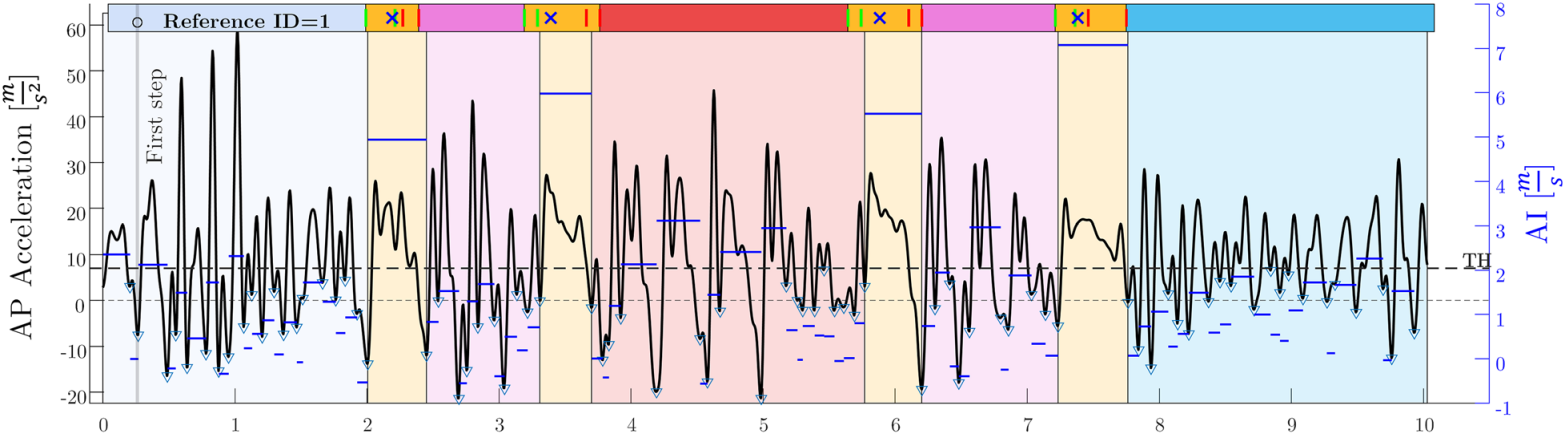
Segmentation of one test sample using reconstructed AP acceleration signal. T_MA is indicated by dotted horizontal line. Blue horizontal line shows the acceleration impulse between each local minimum; blue triangles show all the local minimum; timeline at the top comes from video reference with the blue crosses showing the instance of cone touch, the green and red vertical lines indicating heel strike and toe off, respectively. This timeline is shaded darker than the segments below, to highlight its role as reference from video.

Four transient phases, corresponding to each of COD segments, were visible with a simple visualization of the AP acceleration signal (Fig. 2). For transient signals, wavelet analysis enables reconstruction of the signal with minimal phase shift and loss of information. Wavelet decomposition also preserves features such as a discontinuity better than spectral analysis^27^. Some studies have also shown that the magnitude of variables (such as vertical acceleration while running) strongly depends on the filter cut-off frequency^28^. Therefore, wavelet decomposition within a range of 0.5–15 Hz was used to approximate the shape of AP acceleration signal. The mean value of the AP acceleration (zero frequency) was then added back to the wavelet-approximated signal to synchronize it with the original signal. Local minima below a specific threshold (${T}_{MA}$ in Eq. 1), with prominence greater than 15 ms^−2^, were detected using the MATLAB *peakfinder* algorithm. ${T}_{MA}$ was chosen as a function of the average peak value (resulting from the ground contact):

1 $${T}_{MA}= \frac{1}{4}\times \frac{\sum (local\ max.\ peak\ value)}{number\ of\ local\ max.\ peaks}$$

The AP acceleration between each detected local minima was then numerically integrated using the *cumtrapz* function to approximate the change of velocity during a foot stance. This change in velocity during the COD motion is referred to as the *acceleration impulse* (AI). As we see in Fig. 2, the acceleration impulses (in blue) are around 5–7 ms^−1^ during COD and between 1 and 3 ms^−1^ during displacement phases. Four pairs of the detected minima with the maximum AI values were selected as the COD segments. Since the order of the 90° and 180° COD segments and the timestamps of the four pairs of minima were known, the COD type was also classified.

#### Micro-analysis

The COD detection algorithm presented in the macro-analysis section is used as a first approximation for the beginning and the end of the COD events. The micro analysis algorithm was subsequently used for a more precise detection of the start/end of the COD segments. The key event during each COD is typically defined using the last step before shifting to the new direction, called ‘final foot contact’ (FFC)^29^, which is indicated as the second heel-strike (for example, L_in_ in COD 2) in Figure S1. However, research has also shown the importance of the step preceding the FFC, especially for sharp turns. This step is called ‘*penultimate foot contact*’ (PFC), indicated as the first heel strike (for example, R_in_ in COD 2) in Figure S1, and is used to brake prior to the COD^29,30^. For CODs greater than 60°, it is recommended to brake strongly during the PFC, thus making it important^8^. For the micro-analysis, five candidate methods were developed based on the observation of the signals, and the obtained error over 6 participants was used to evaluate these methods:

- M0: The COD detection from macro analysis (Fig. 2) was used. Start and end of the COD were the local minima between the largest AP acceleration impulse.
- M1: Within the selected local minima, a smaller wavelet range (0.5–5 Hz) was applied, and the resultant local minima were selected as the start and end events.
- M2 (only for 180° COD): If there was a large peak just before the start detected during macro-analysis, this method advanced the start of COD by a local minimum. The goal was to shift the detected start from the second HS to the first, i.e., PFC. The end of COD, which was detected during macro-analysis, was shifted forward by a local minimum if the detected minima (end) was less than 0. This was done to shift the endpoint to the second TO.
- M3: First point on the acceleration norm signal with values less than g was found, which occurred immediately near the approximate COD start/end found with the macro analysis. Acceleration norm < g indicates the flight phases, which occurs immediately before the first HS and after the second TO.
- M4: For the first 90° COD (COD 1), the end of COD detected from the macro-analysis was moved forward by one local minimum if the detected end was immediately followed by a peak. This peak indicates the second TO, which occurs at the end of the COD 1.

#### Total completion time estimation

It is possible to estimate total completion time by integrating GNSS ground speed (${v}_{GNSS}(t)$) in the last displacement phase. We consider estimated timestamp for the end of last COD (${\widehat{t}}_{COD4end}$) and estimated total completion time (${T}_{estc}$) as the lower and upper bounds of an integral, which should be equal to the last displacement phase (${L}_{DP5}$) minus the arm reach (${L}_{AR}$). We assumed ${L}_{AR}=1.42 m$ based on the anthropomorphic measurements data for 95th percentile of adult males^31^. We can solve Eq. (2) using numerical integration to obtain an estimation for total time:

2 $${L}_{DP5}-{L}_{AR}={\int }_{{\widehat{t}}_{COD4end}}^{{T}_{estc}}{v}_{GNSS}(t)dt$$

#### Validation

For each micro-analysis method, the error (${\varepsilon }_{s}$ for start and ${\varepsilon }_{e}$ for end) in ms between detected COD start (${T}_{ests}$) and end (${T}_{este}$) timestamps with respect to the corresponding reference timestamps (${T}_{refs}$ and ${T}_{refe}$) were obtained from the video labels:

3 $${\varepsilon }_{s}= {T}_{refs}- {T}_{ests}$$

4 $${\varepsilon }_{e}= {T}_{refe}- {T}_{este}$$

If there is a shift due to the assumption of the first step as a starting point, it should be observable across COD events for a within-subject observation. Here, shift is considered when the mean of the error has a higher magnitude than the standard deviation (SD), with positive sign considered as positive shift and vice-verse for negative shift. To investigate this shift, we investigated the mean ± S.D. of ${\upvarepsilon }_{\mathrm{s}}$ and ${\upvarepsilon }_{\mathrm{e}}$ for each participant across all CODs. Based on the start and end points, duration of each segments (4 CODs and 5 DPs) was also computed, as it is an important metric for analysis of the performance during the T-test^12^. For the durations, the absolute error (${\varepsilon }_{da}$) in ms and relative error (${\varepsilon }_{dr}$) in % were computed from estimated (${D}_{e}$) and reference (${D}_{r}$) durations. The error for total completion time (${\varepsilon }_{dc}$) was also computed, based on the difference between the duration recorded by the photocell (${T}_{refc}$) and the one estimated (${T}_{estc}$) using Eq. (2). Finally, the Bland–Altman plot^32^ was utilized to investigate any correlation between the errors (${\varepsilon }_{s}$, ${\varepsilon }_{e}$, and ${\varepsilon }_{dc}$), the detected events (${T}_{refe}$ and ${T}_{este}$) and estimated completion time (${T}_{refc}$ and ${T}_{estc}$). Equations (5), (6), and (7) show the computation of the errors. The distribution of ${\varepsilon }_{s}$ and ${\varepsilon }_{e}$ was tested for normality using the Kolmogorov–Smirnov test and the distribution of ${\varepsilon }_{dc}$ was tested using Shapiro–Wilk test, according to published recommendations for normality tests^33^.

5 $${\varepsilon }_{da}= {D}_{r}- {D}_{e}$$

6 $${\varepsilon }_{dr}= \frac{{D}_{r}- {D}_{e}}{{D}_{r}}\times 100 \%$$

7 $${\varepsilon }_{dc}= {T}_{refc}-{T}_{estc}$$

### Metrics for performance in COD test

While the proposed method for instrumenting the agility test can provide additional data during the COD, we used existing performance metrics from literature^8,12,14,34^. Among a larger set of metrics that can be estimated using a combination of force plate and motion capture systems, we considered those that can be estimated using the current sensor setup for further analysis. We selected the best’ and ‘worst’ groups of five participants each to investigate the relationship between the metrics and athlete’s performance. The ‘best’ and ‘worst’ performance was assessed with the total completion time, as this is the standard metric used to evaluate the T-test. The total completion time of the test is the standard performance metric, which comes from the photocells. The duration of each COD and displacement phases was used to gain insight into the potential weakness/strength for different movements. Finally, the total cutting time, which is the sum of the approach phase (DP before COD) duration, the COD duration, and the exit phase (DP after COD) duration, was computed to assess the performance of a single COD. Pearson correlation^35^ between total cutting time for each COD and the total completion time was evaluated to investigate the relevance of total cutting time as a performance metrics. Additionally, the individual durations of all nine segments, and the total cutting time for each COD were visually compared for the five athletes with highest and lowest total completion times. This provides an insight into potential discriminating factors in performance.

### Ethical approval

The study protocol was reviewed and approved by EPFL Human Research Ethics Committee (HREC 053-2020) and conducted according to the declaration of Helsinki. The patients/participants provided their written informed consent to participate in this study.

## Results

The total test completion time (mean ± std) based on photocells was 9.11 ± 0.35 s and 9.32 ± 0.35 s for the two trials. Details on the completion time and an illustration of the video labelling for 8 athletes are presented in Figure S2 in Supplementary materials. Considering this video data as the reference, results for the detection of COD start/stop and the performance metrics are presented in this section. The profile for the speed, obtained using the GNSS receiver, is presented in the Figure S3 in supplementary materials.

### Detection and duration of COD

The proposed method was capable of detecting all the phases of COD and distinguishing between the 90° and 180° CODs. With the detected first step assumed as the start (t = 0 s), T-test timelines for four participants are presented in Figure S4. We observed a simultaneous positive shift in the detected COD start (${\varepsilon }_{s}$) and end (${\varepsilon }_{e}$) in only three participants and a simultaneous negative shift in only four participants (see Table S1). ${\varepsilon }_{s}$ and ${\varepsilon }_{e}$ for each COD from the four micro-analysis methods are presented in Figure S5. Based on the lowest error, M0 was the best method for detecting the start of COD 1 and the start and stop for COD 4. For COD 2 and COD 3 (180° COD) M2 produced the lowest error for both start and stop. Finally, for detecting the end of COD 1, M4 led to the best results. The errors resulting from the combination of the above-mentioned best methods are presented in Table 1. The maximum relative errors were less than 7% and 15% for the estimation of phase and COD duration, respectively.

**Table 1:** Mean ± standard deviation of estimation error and (%) for each displacement phase (DP) and change-of-direction (COD) across all participants.

	DP 1	DP 2	DP 3	DP 4	DP 5
DP duration					
${\varepsilon }_{da}$ (ms)	− 3 ± 53	− 2 ± 42.2	− 20 ± 68.2	19 ± 50.4	− 135 ± 142.7
${\varepsilon }_{dr}$ (%)	− 0.2 ± 3.13	− 0.3 ± 5.16	− 1.1 ± 3.65	2 ± 6.01	− 6.7 ± 6.93

	COD 1	COD 2	COD 3	COD 4	
COD duration					
${\varepsilon }_{da}$ (ms)	− 10 ± 51.4	25 ± 72.3	2 ± 69.8	5 ± 59.6	
${\varepsilon }_{dr}$ (%)	− 2.7 ± 14.08	3.5 ± 13.21	− 0.2 ± 12.32	1.3 ± 14.47	
COD event					
${\varepsilon }_{s}$ (ms)	− 2.5 ± 53	− 12.9 ± 55.3	− 8.7 ± 73.7	11.4 ± 63.9	
${\varepsilon }_{e}$ (ms)	− 12.7 ± 59.3	16.5 ± 77.9	− 7.4 ± 75.4	16.5 ± 69.6	

**Figure 3:**
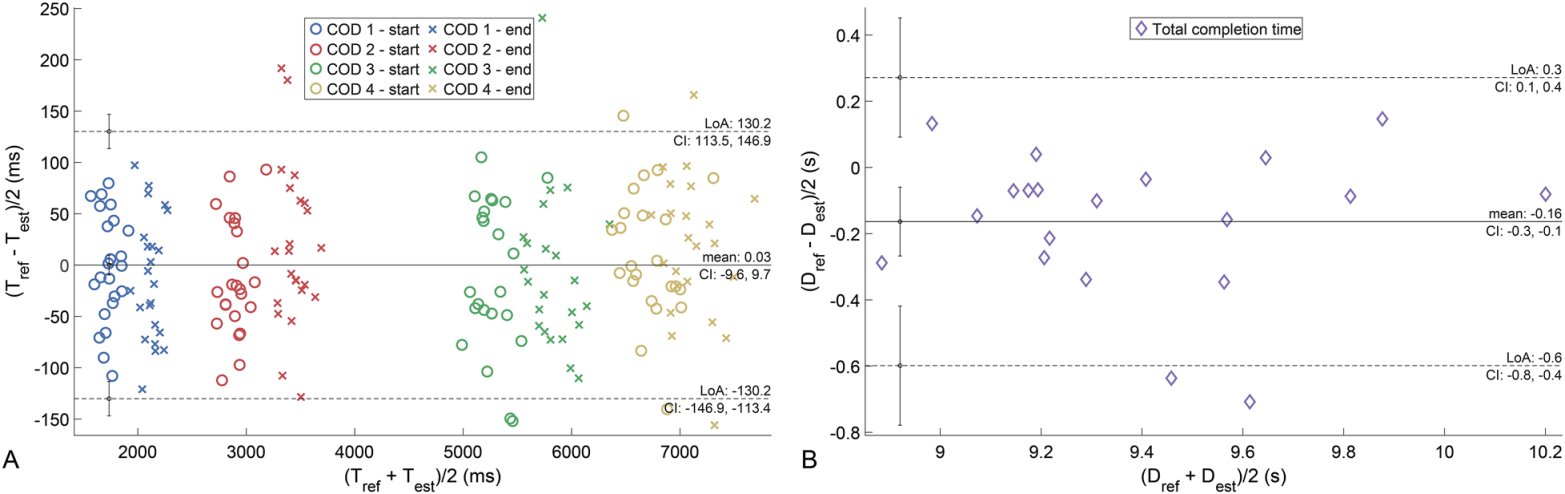
Bland–Altman plot for detected COD start/end events and total completion time. Results of the combination of best methods are presented here for COD detection, *LOA (ms)* limits of agreement and *CI* confidence interval.

Figure 3A presents the Bland–Altman plot for the estimation of the COD start/end events using the combination of best methods presented above. T_est_ represents the timestamp of the detected event using the proposed method, while T_ref_ represents the timestamp for the same event obtained using the video data. The 95% limit of agreement (LOA) of the proposed method was between − 130.2 and 130.2 ms and the mean difference is 0.0 ms. The Bland–Altman plot did not show a systematic trend for the estimation errors. The error ${\varepsilon }_{dc}$ (mean ± standard deviation) in the estimated total completion time was − 0.16 ± 0.22 s. Since the GNSS data for three participants was not recorded appropriately, this result was computed over twenty participants. The 95% LOA was between − 0.6 and 0.3 s, while the Bland–Altman plot for this error does not present a systematic trend for the estimation error.

### Performance metrics

**Figure 4:**
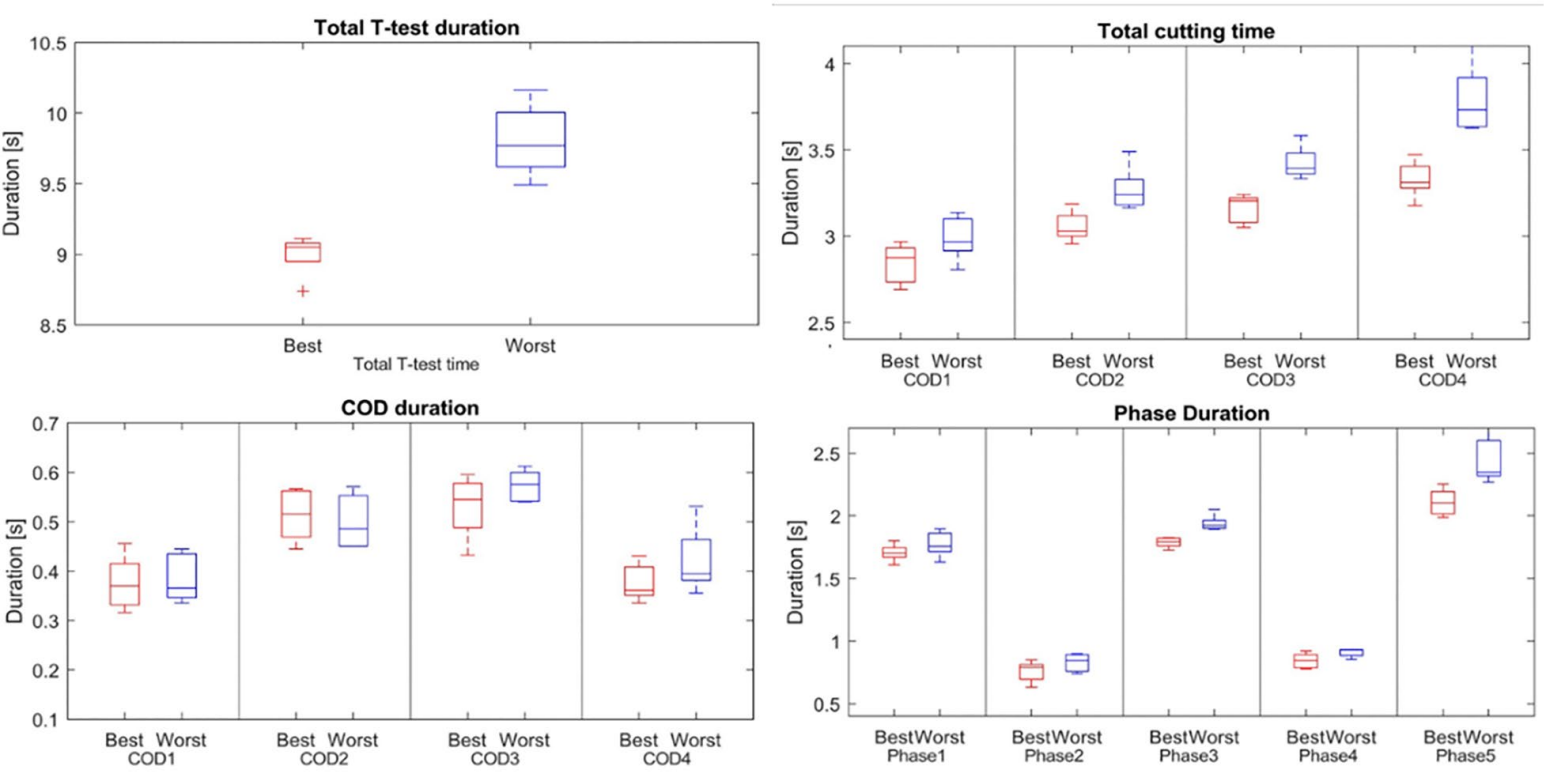
Comparison between five best (red) and worst (blue) participants in terms of results. Best and worst groups comprised of five participants each with lowest and highest total completion times on the test, respectively.

It was found that total cutting time of each COD was correlated to total completion time (Figure S2 in the supplementary material), suggesting that this performance metric indeed reflects the general T-test performance of elite athletes. The Pearson correlation coefficient for each COD was as follows—COD 1: 0.58 (p < 0.01), COD 2: 0.62 (p < 0.001), COD 3: 0.70 (p < 0.0001), and COD 4: 0.78 (p < 0.0001). The duration of COD did not correspond to the duration of the test, with fastest five and slowest five athletes spending similar amount of time during COD (Fig. 4). The best and worst groups spent similar time in CODs (Fig. 4), but fast athletes were generally faster during the displacement phases. The difference in performance seems to arise more from the long shuffle to the right and the backward sprint than from other displacement phases. Furthermore, all four total cutting times, as seen with the correlation, can also differentiate well between the two performance groups.

## Discussion

The labelling accuracy of each event is around 3 frames, which corresponds to an error of approximately 50 ms for a camera with 60 Hz frame rate. The S.D. of the error for detection of all COD events (Fig. 3) was 66 ms, which is close to this expected range of 50 ms. It is also in the similar range as other studies, which utilized IMU and video reference to segment motion phases during highly dynamic activities^26^. In Fig. 4, we can see a minimal bias in the COD1 and COD4 (90° angle) events detection, whereas the 180° COD are relatively more biased. This is because the method M0, directly based on the macro-analysis, sometimes detects the second step in and the first step out of the 180° COD. This does not happen in 90° COD because almost all the braking motion happens during the first step in. In method M2, if a large peak is detected just prior to the COD start reference from macro detection, the COD start point is moved before this peak. For the COD end, if the AP acceleration is bigger than 0, the end is pushed to the next local minimum smaller than 0. This method thus allows to get the first step in if the second heel strike is detected by the macro analysis and the second toe off if the first was previously detected by the macro analysis. Therefore, this method provides the best result (Figure S5 in supplementary materials) for the COD 2 and COD 3, with the 180° rotation. Method M4 improves the detection of the end of the first 90° COD (COD 1), by using the same principle as method M2 of shifting the detected end by one local minimum.

While one cause of the variation in the S.D. of COD event detection between 53 and 77.9 ms (Table 1) was the low frame rate of the reference video, another important could be the COD technique used by the athletes. The athletes who crossed legs during COD or the side shuffle were identified from video but not eliminated, since this phenomenon is difficult to address during testing in the field^36^. To decelerate and then accelerate the body in the new direction, the COD motion implies an important antero-posterior (AP) and medio-lateral (ML) acceleration impulse on the ground and on the trunk^37,38^. These AP and ML impulses were clearly observable in the IMU signal and were enclosed by the labeled step events defining the change of direction. The detected AP acceleration impulse (Fig. 2) was positive for each COD, even though the COD angle and technique exhibit large differences on the video recordings. This is likely because of the positive reaction force in AP direction created by the braking force on the foot of the athlete. For the same reason, local minima before high peaks on the AP acceleration align with the impact of each step during every motion phase (Fig. 2), despite the different motion techniques—shuffle, sprint forward, and sprint backward.

The error in total completion time (− 0.16 ± 0.22 s) was close to the frequency of the GNSS unit (0.1 s) and the difference between the mean values (0.21 s) of the completion times for the two trials with same instructions, despite the highly homogeneous population. This indicates acceptable performance for repeated measurements for athletes through the season. The proposed method overestimates the test duration due to the photocells being positioned slightly ahead of the cone, thereby causing the athletes to pass the photocell earlier than the assumed distance of 9.14 m at the end. The assumption of the arm reach distance (${L}_{AR}$) of 1.42 m was based on data for 95th percentile males and introduces precision error since athletes have varying arm reaches. The error on phase duration has an absolute mean error smaller than 25 ms for all displacement phases, except for the backward run phase (− 135 ms). This larger error comes from both the error on the total completion time and the choice to synchronize the signal and the video with the first step. Some athlete crossed the starting line before their first step and others after. As the first step was considered as the beginning of the test in the IMU signal analysis, this creates an error on the end time of the test only. As seen in (Table 1), the mean relative error for motion phases is around 5%, but the standard deviation of the relative error for the COD duration is around 15%. This is likely because of the short COD duration (∼ 500 ms), compared to the precision of the general method (∼ 50 ms).

The duration of CODs did not correlate with the duration of the test, with fast and slow athletes spending similar time during COD (Fig. 4), despite it representing roughly 20% of the total duration. Conversely, correlation between the total test completion and the total cutting time for each COD reflects the influence of cutting performance on the T-test performance for elite athletes. This observation confirms the fact that T-test assesses a mix of functional capacity in each displacement phase and that the difference in performance in elite athletes comes from each part of the test^2,8,34^. Therefore, it seems less useful to "rush" the COD movement to save time on the T-test, but better to focus on the speed during the displacement phases. However, this claim needs to be validated over a larger sample of athletes with varied skill levels. The COD duration alone can also be used as a measure of performance, and although it cannot be assessed in the traditional T-test scoring method, it can be assessed using the proposed method. Differences in the 'best' and 'worst' athlete groups were also highlighted through their speed profile (see Figure S6 in supplementary material); faster athletes show a higher speed drop just before or during the COD’s and a stronger acceleration after 180° CODs. This suggests that the ability to accelerate during shuffle phases may be key to T-test performance. Interestingly, the minimum speed during the first 90° COD is lower in faster athletes. This suggests that the ability to decelerate quickly is more important than absolute speed during COD, which is consistent with literature^11^. These observations underline the fact that the ability to perform a COD speed test depends not only on the COD itself, but also on the ability to accelerate and decelerate during the displacement phases, reflecting the plyometric qualities of the athletes. To further investigate these abilities in the field, additional metrics such as approach and exit speed, minimum speed, peak absolute acceleration, and acceleration impulse can be estimated during each COD^8,14,34^.

### Limitations and future work

The synchronization between video and IMU can be improved by asking the athlete to do an easily detectable movement (e.g., standing jump) before the test. A better way to synchronize is to use a simultaneously detectable electronic pulse or obtain the precise GMT/UNIX timestamp. Furthermore, using a higher frame rate for the reference video camera will help to have a better resolution and therefore improve the estimation of the accuracy of the proposed methods. Adding IMU on the feet would allow us to differentiate right and left leg ground contact time (or PFC from FFC) and enable further investigation into how the impact on the ground is transferred to the trunk. However, the use of a higher sampling frequency (more than 462 Hz) is recommended in order to reduce errors in the estimate of GRF and ground contact time^39^. Adding an IMU on the sacrum could help to get data more related to the lower limb and independent of the compensatory movement of the trunk. However, to detect COD precisely, the single IMU on the back is sufficient. The single IMU on the trunk, worn in a vest, is commonly used in existing testing protocols and in competitive games by soccer players; therefore, the application of the proposed methods is easier in practice.

The micro-analysis algorithm uses external inputs such as relative thresholds, frequency range of reconstruction, or time window around COD. Therefore, a sensitivity analysis can potentially improve the robustness of the algorithm. The maximum acceleration was taken on the 15 Hz wavelet-reconstructed signal to avoid unrealistic peak acceleration values. As the range of frequency for this reconstructed signal affects the maximum acceleration value^40^, the exact correlation between maximum GRF and reconstructed acceleration should be clarified. The sign of ML angular speed changes  during COD because of the straightening of the chest after the athlete touches the cone. Because trunk motion correlates with COD cutting performance, ML angular velocity profile could provide a complementary method to detect COD. It may also provide a way to distinguish between the eccentric and concentric COD phases. Additionally, automated pattern recognition algorithms could be implemented to recognize the signal shape for each COD angle (90°, 180°, etc.) to enable COD analysis during match play^41^. For detecting the end of the T-test, the validity of the GNSS ground speed should be inspected due to its low sampling frequency (10 Hz). Furthermore, this limitation can be addressed by fusing the IMU and GNSS information, wherein the IMU can provide the necessary sampling rate and the GNSS speed can be used to correct the drift in the IMU-based speed^25^. An important source of variability in movement mechanics is the athlete population, with females demonstrating significantly less peak hip abduction than did males during COD maneuvers^42^. Thus, the proposed methods should be at least validated separately for female soccer players.

## Conclusion

The proposed method can be used to determine the duration of the five displacement phases and detection of the COD events using a single trunk-worn GNSS-IMU unit during a T-test. It uses the large anteroposterior change in momentum caused by the braking and acceleration phases of the COD to detect them. The Bland–Altman analysis for all COD events detected in the T-test shows a mean error of − 0 ± 66 ms and a 95% confidence interval of ± 130 ms (3.9%), compared to reference data from video camera. Thus, the T-test were successfully divided into 9 phases, allowing coaches to better understand the athletes’ technique and physical qualities during each displacement phase and COD types, and may prove to be a valuable performance evaluation tool for coaches. For example, the observation that total cutting time is correlated to the total completion or that the displacement phase duration could differentiate between the best/worst five performers, could be useful for identifying athletes’ strengths and weaknesses. Furthermore, asymmetrical performance between displacement in the right and in the left detection can be highlighted, which can potentially provide information about the position in the field where the player will perform the best. Right and left asymmetries during COD duration could be sign of fatigue in one of the knees and/or an asymmetry in the strength of the muscles used for braking. This information can help the coach and the strength and conditioning staff to develop a more personalized training and rehabilitation program.

## Supplementary Information

Supplementary Information.

## Data Availability

The data is available from the corresponding author on reasonable request.

## Abbreviations

COD: Change-of-direction
DP: Displacement phase
GNSS: Global navigation satellite system
IMU: Inertial measurement unit
ACL: Anterior cruciate ligament
ROM: Range of motion
COM: Centre of mass
PFC: Penultimate foot contact
FFC: Final foot contact
AI: Acceleration impulse
AP: Anteroposterior
ML: Mediolateral
CC: Craniocaudal
LOA: Limits of agreement
g: Acceleration due to gravity (9.81 ms^−^^2^)
