# Supplementary material for: In-field assessment of change-of-direction ability with a single wearable sensor
Source: Sci Rep. 2023 Mar 18;13:4518. doi: 10.1038/s41598-023-30773-y (PMC10024719; doi:10.1038/s41598-023-30773-y)
Supplement: Supplementary file 1 — Supplementary Information. [file 41598_2023_30773_MOESM1_ESM.pdf]

Supplementary materials: In-field assessment of change-of-direction ability with a single wearable sensor

Labelling of video data

Assigned events for each COD are depicted in the figure below. Time frames of each test were recorded using Kinovea and then imported into MATLAB. Each test data was manually cut in a 15-second window (the duration of the T- test is typically around 10s) for subsequent algorithm development and validation.

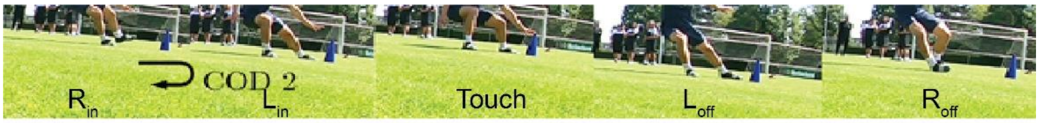

Figure S1. Event labels for COD 2 segment to be used as reference data; COD 1 and COD 4 involve 90° turn with a sidestep, COD 2 and COD 3 involve 180° turn with a split step, R_{in} in: right foot heel strike, L_{in}: left foot heel strike, R_{off}: right foot toe-off, L_{off}: left foot toe-off

For each T-test, the results of video labelling consist of 27 frames corresponding to one event, with the most important events (test start, COD start/stop, etc.) shown below. 20 Segmentation of the T-test based on video labelling for 8 of the 23 athletes. The total completion time of the T-test (based on video) are given on the right of split T-test.

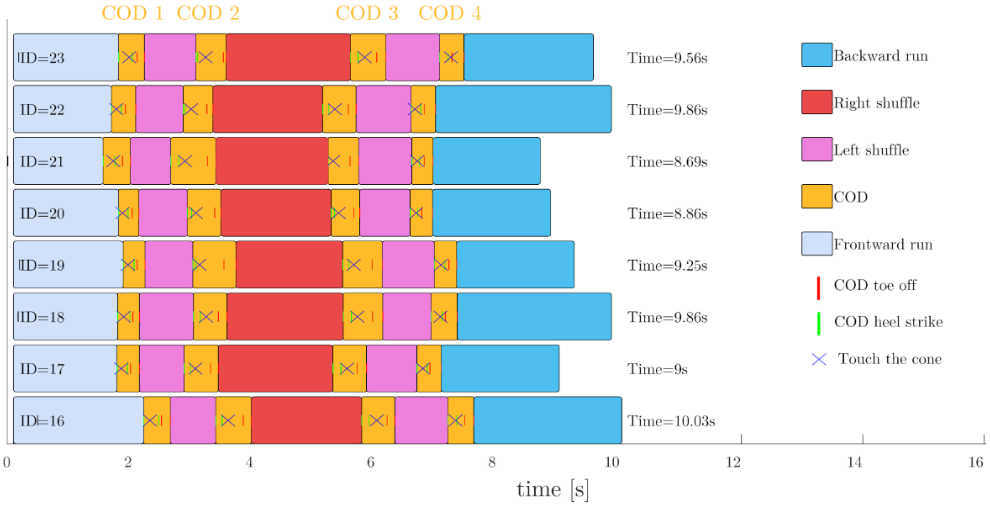

Figure S2. Segmentation of the T-test based on video labelling for 8 of the 23 athletes. The total completion time of the T-test (based on video) are given on the right of split T-test

Speed profile

The profile for mean ± S.D. of speed is presented below, with solid and dashed lines indicating the mean and S.D., respectively. The boxes show the detected mean start and end of the segments. It can be observed that the speed indeed reaches its lowest values during the 180 deg COD segments, while the speed reaches its peak closer to the end of the first displacement phase that involves running forward. Furthermore, we can observe that participants did not accelerate as strongly in the last displacement phase as during the first one.

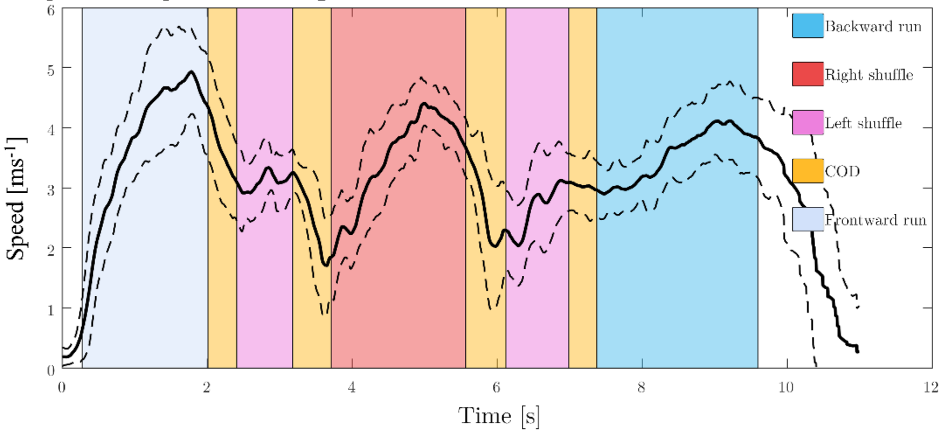

Figure S3. Mean (solid line) and S.D. (dashed lines) for the speed obtained using the GNSS receiver during the T-test for all 23 athletes. Colored rectangles show phases based on mean event time found with the segmentation algorithm, with standard deviation of these events omitted for the sake of clarity

Detection and duration of COD

The proposed method was able to detect all the COD phases and differentiate between the 90 deg and 180 deg CODs. With the detected first step assumed as the start (t = 0 s), T-test timelines for four participants are illustrated in Figure S2.

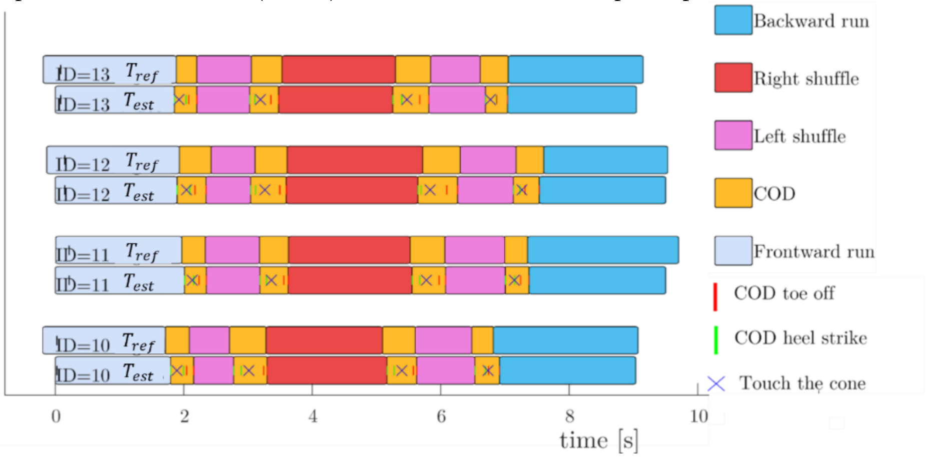

Figure S4. Event timestamp from video and algorithm for four T-tests. Timelines are aligned using the first step and the end of the signal timeline is the duration from the first step to the photocell end time. Results for method M0 are shown

ε_s and ε_e for each COD from the four micro-analysis methods are presented in Figure S3. Based on the lowest error, M0 was the best method for detecting the start of COD 1 and the start and stop for COD 4. For COD 2 and COD 3 (180 deg COD) M2 produced the lowest error for start and stop. Finally, for detecting the end of COD 1, M4 led to the best results.

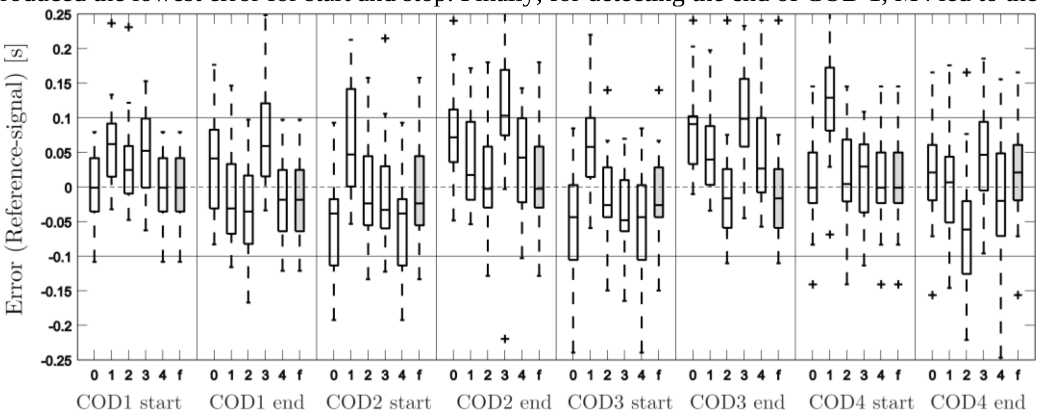

Figure S5. Box plot of ε_s and ε_e for COD detection for each micro analysis method. The "f" boxplot shows the best method for each event detection, among the five methods.

A typical pattern was observed for fast and slow athletes (see below); faster athletes show a higher speed reduction just before or during the COD's. Also, a stronger acceleration after 180° cuts can be observed.

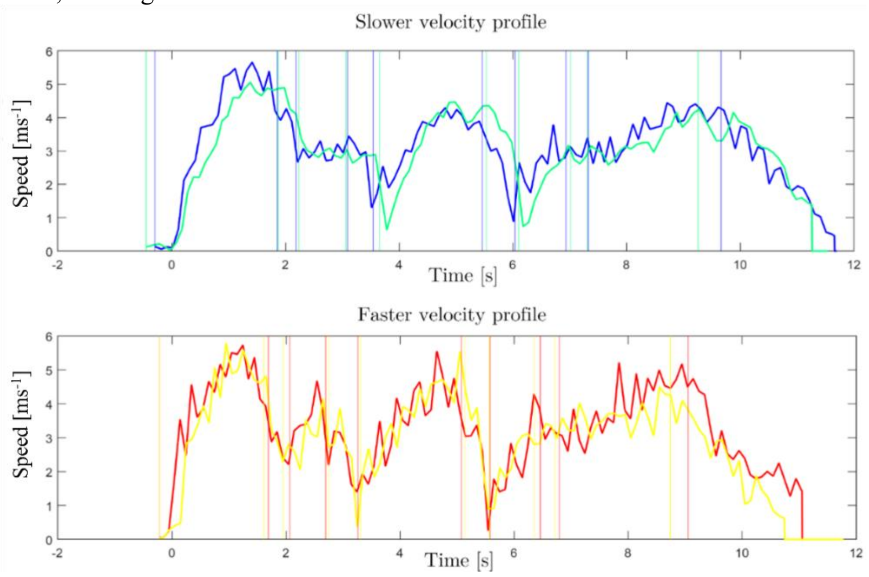

Figure S6. Speed profile during the T-test for two fastest and slowest athletes. Vertical lines show the key events during the T-test (start and end of the COD, end of the T-test). The speed profiles are aligned using the first step

Errors for COD start and end for each athlete are presented in the table below. A simultaneous positive shift in the detected COD start (ε_s) and end (ε_e) was observed in three participants and a simultaneous negative shift in four participants.

Table S1. Mean ± S.D. of estimation error for each participant across all CODs, $\boldsymbol{\varepsilon_s}$ = COD start, $\boldsymbol{\varepsilon_e}$ = COD end. The participants with either a simultaneous positive or negative shift in $\boldsymbol{\varepsilon_s}$ and $\boldsymbol{\varepsilon_e}$ are indicated in bold.

Id	COD 1		COD 2		COD 3		COD 4		Mean ± SD	
	ε_s (ms)	ε_e (ms)	ε_s (ms)	ε_e (ms)	ε_s (ms)	ε_e (ms)	ε_s (ms)	ε_e (ms)	ε_s (ms)	ε_e (ms)
**1**	**13.2**	**58.2**	**23.5**	**24.5**	**48.8**	**100.5**	**20.7**	**11.5**	**26.6±15.4**	**48.7±39.8**
2	-59.0	-18.0	-40.8	15.2	-63.0	72.8	83.5	-26.5	-19.8±69.6	10.9±45.0
3	66.0	77.0	57.0	47.5	77.7	-27.2	-36.3	69.0	41.1±52.3	41.6±47.5
4	-1.5	39.2	20.0	-75.0	38.0	59.3	15.5	46.5	18.0±16.2	17.5±62.2
5	-5.8	5.7	66.7	-87.5	-43.0	43.3	9.2	20.0	6.8±45.6	-4.6±57.4
6	0.8	-14.2	16.7	-53.0	-11.3	15.0	20.7	55.7	6.7±14.8	0.9±46.0
**7**	**-33.5**	**-53.5**	**-93.0**	**-16.8**	**-85.0**	**-39.8**	**-84.8**	**-64.5**	**-74.1±27.3**	**-43.6±20.6**
8	12.0	37.0	38.3	-191.8	26.3	16.2	7.7	-1.7	21.1±13.9	-35.1±105.7
9	-57.5	72.5	49.8	-13.7	-52.2	-15.8	35.0	16.0	-6.2±56.5	14.7±41.1
**10**	**-79.8**	**-69.8**	**-59.5**	**-13.5**	**-67.0**	**-21.3**	**-50.5**	**-95.5**	**-64.2±12.4**	**-50.0±39.2**
11	-43.2	-18.0	-2.0	8.5	-30.0	-9.2	-4.2	-18.5	-19.9±20.1	-9.3±12.6
**12**	**37.0**	**82.7**	**68.2**	**14.5**	**74.0**	**40.0**	**41.2**	**71.2**	**55.1±18.7**	**52.1±30.9**
13	30.5	-3.0	28.0	54.5	43.7	29.0	-74.5	6.0	6.9±54.7	21.6±25.7
14	-8.5	-58.5	-32.8	-62.7	-46.3	-240.8	-145.5	-50.5	-58.3±60.2	-103.1±91.9
15	25.5	65.7	41.0	31.2	152.0	58.3	23.8	-21.0	60.6±61.4	33.6±39.3
**16**	**-69.0**	**-77.3**	**-86.2**	**-180.2**	**-64.5**	**-73.0**	**-87.5**	**-76.8**	**-76.8±11.8**	**-101.8±52.3**
17	70.7	121.0	112.2	107.8	103.8	65.0	1.0	-79.0	71.9±50.6	53.7±91.6
**18**	**90.2**	**41.2**	**97.3**	**128.5**	**149.5**	**110.2**	**140.5**	**156.0**	**119.4±30.0**	**109.0±48.9**
19	108.0	-27.0	19.0	-60.5	26.0	46.0	-92.5	-165.7	15.1±82.3	-51.8±88.0
20	18.8	25.0	26.3	37.0	42.0	4.5	-34.3	-48.7	13.2±33.1	4.4±37.9
21	47.7	83.7	38.7	19.2	47.2	72.2	42.5	-96.5	44.1±4.3	19.7±82.4
22	-37.8	18.5	-45.8	-20.5	-105.0	-59.5	-48.2	-47.8	-59.2±30.9	-27.3±34.6
**23**	**-67.2**	**-97.3**	**-46.0**	**-93.0**	**-61.5**	**-75.5**	**-44.5**	**-39.5**	**-54.8±11.3**	**-76.3±26.3**
